# Is Telomere Length Socially Patterned? Evidence from the West of Scotland Twenty-07 Study

**DOI:** 10.1371/journal.pone.0041805

**Published:** 2012-07-23

**Authors:** Tony Robertson, G. David Batty, Geoff Der, Michael J. Green, Liane M. McGlynn, Alan McIntyre, Paul G. Shiels, Michaela Benzeval

**Affiliations:** 1 Medical Research Council’s Social & Public Health Sciences Unit, Glasgow, United Kingdom; 2 Institute of Cancer Sciences, MVLS, University of Glasgow, Glasgow, United Kingdom; 3 Department of Epidemiology & Public Health, University College London, London, United Kingdom; McGill University/Douglas Mental Health Univ. Institute, Canada

## Abstract

Lower socioeconomic status (SES) is strongly associated with an increased risk of morbidity and premature mortality, but it is not known if the same is true for telomere length, a marker often used to assess biological ageing. The West of Scotland Twenty-07 Study was used to investigate this and consists of three cohorts aged approximately 35 (N = 775), 55 (N = 866) and 75 years (N = 544) at the time of telomere length measurement. Four sets of measurements of SES were investigated: those collected contemporaneously with telomere length assessment, educational markers, SES in childhood and SES over the preceding twenty years. We found mixed evidence for an association between SES and telomere length. In 35-year-olds, many of the education and childhood SES measures were associated with telomere length, i.e. those in poorer circumstances had shorter telomeres, as was intergenerational social mobility, but not accumulated disadvantage. A crude estimate showed that, at the same chronological age, social renters, for example, were nine years (biologically) older than home owners. No consistent associations were apparent in those aged 55 or 75. There is evidence of an association between SES and telomere length, but only in younger adults and most strongly using education and childhood SES measures. These results may reflect that childhood is a sensitive period for telomere attrition. The cohort differences are possibly the result of survival bias suppressing the SES-telomere association; cohort effects with regard different experiences of SES; or telomere possibly being a less effective marker of biological ageing at older ages.

## Introduction

Inequalities in health are not only present between the richest and poorest members of society, but there is also a social gradient in life expectancy, mortality and morbidity across the full socioeconomic spectrum [1]. Increased exposure to physical and psychological insults, along with more health-damaging behaviours, has the potential to increase cellular and genomic damage, thereby accelerating biological ageing (ageing at the cellular and organ level that is affected by genetic, metabolic and environmental factors) [2]. People in more disadvantaged circumstances, where these insults are more prevalent [3], would therefore be expected to be ‘biologically’ older than their more affluent counterparts of the same chronological age. It has been hypothesised that this accelerated ageing in those with lower socioeconomic status (SES) could be a mechanism that increases the risk of premature mortality and developing chronic diseases such as cancer and cardiovascular disease earlier in life [4].

Telomeres are protective structures present at the ends of chromosomes that typically erode over time to protect against irreversible chromosomal damage [5]. This progressive reduction in telomere length has made telomeres an appealing, widely utilised measure of an individual’s biological age [6]. Telomere length has been shown to be associated with ageing-related diseases such as dementia [7], chronic kidney disease [8] and some cancers [9]–[10], as well as mortality [11]. If socioeconomic disadvantage does lead to cellular damage and more rapid biological ageing, this should be reflected in the form of shorter telomeres [2]. Alternatively (and possibly additionally), certain diseases may shorten telomeres, mirroring the patterns of socioeconomic status in health rather than causing shorter telomeres. Due to the cross-sectional nature of most of the literature regarding SES and telomere length, it is not possible to determine causation.

The evidence for a relationship between SES and telomere length is mixed: some investigators find associations between disadvantaged SES and shorter telomeres [12]–[15], others the opposite [16]–[17], while many report non-significant associations [18]–[30]. Indeed, depending on the SES marker utilised, many studies find a mix of both positive and null results [4], [31]–[34]. Against this background of discordant findings, the aim of the present study was to examine the associations between SES and telomere length in three age cohorts from the West of Scotland Twenty-07 Study utilising a comprehensive range of SES measures across key periods of the lifecourse. By focusing on the key periods of contemporary life, childhood, the education years (encompassing both childhood and early adulthood) and on accumulation across these stages, our aim was to identify general patterns of SES associations with telomere length at different ages with respect to different lifecourse models. It has been hypothesised that early life/childhood may represent a more sensitive period for telomere attrition than adulthood [34]. In addition, it could be that accumulated exposure to lower SES throughout the lifecourse is associated with greater telomere attrition. If childhood is a sensitive period we would expect to find that the childhood SES and education measures would be more consistently and strongly associated with telomere length compared to contemporaneous SES measures. If telomere length is most affected by repeated insults due to cumulative SES, we would expect the accumulation measures to show the strongest associations. As the associations between SES and telomere length are not well understood, we have used both continuous and categorical measures of SES to assess the strength of a gradient across the whole SES hierarchy, as well as comparing extreme SES categories. To our knowledge, this is the most comprehensive study to date to explore these associations.

## Materials and Methods

### Study Sample

The West of Scotland Twenty-07 Study is a community-based, prospective cohort study designed to investigate the social processes that produce or maintain inequalities in health. The study has been described in detail previously [35]–[36]. In brief, Twenty-07 consists of three cohorts recruited at the (approximate) ages of 15 (‘1970s cohort’), 35 (‘1950s cohort’) and 55 years (‘1930s cohort’) at study baseline in 1987 (wave 1). Data, including blood samples at wave 5 (2007/8), were collected by trained nurses in the homes of the study participants. Ethical approval for the baseline study was granted in 1986 by the GP Sub-Committee of Greater Glasgow Health Board and the ethics sub-committee of the West of Scotland Area Medical Committees. Wave 5 was approved by the Tayside Committee on Medical Research Ethics. Informed, written consent was obtained from all respondents at each wave of the study. For the 1970s cohort at wave 1 (when aged 15), written consent was obtained from parents/guardians and the respondents.

While the baseline sample totalled 4510 (2414 women), the eligible sample had been reduced to 3861 by the start of wave 5 (649 deaths), with 2604 agreeing to take part (75%) [37]. Of these, 2310 respondents consented to blood being taken and 2185 respondents (1191 women, one outlier excluded) were measured for telomere length. Thus, the analytical samples were 775 for the 1970s cohort, 866 for the 1950s cohort and 544 for the 1930s cohort. The sample has become less representative of the baseline population over time, given that those with lower SES and poorer health were more likely to have dropped out or died before telomere length analysis at wave 5. However, these patterns of selective death/drop-out were not equal across the three cohorts. For example, those living in more affluent areas at wave 1 were less likely to have died in the 1950s (Odd Ratio (OR) = 0.458, 95% CI = 0.297; 0.709, *P*<0.001) and 1930s cohorts (OR = 0.670, 95% CI = 0.543; 0.826, *P*<0.001) compared to those in more deprived areas, but this was not the case for the 1970s cohort (OR = 0.891, 95% CI = 0.402; 1.975, *P = *0.776). Table S1 contains the numbers of respondents who dropped out, died or gave a telomere sample by wave 5. Tables S2 & S3 contain the full results for the risk of drop-out (Table S2) or death (Table S3) by wave 5 given various socioeconomic and health characteristics at baseline. In order to adjust for differences in drop out, inverse probability weights have been employed to weight the analysis sample to represent the baseline sample still alive. The implications of survival bias are discussed below.

### Telomere Length Determination

DNA was extracted from peripheral blood leukocytes using the Maxwell® automated purification system (Promega, WI, USA). Telomere length determination was performed blindly in triplicate using a Roche Light Cycler LC480, using a single-copy gene amplicon primer set (acidic ribosomal phosphoprotein, 36B4) and a telomere-specific amplicon primer set [38]. Relative telomere length was estimated from the Cycle-threshold (Ct) scores using the comparative Ct method after confirming that the telomere and control gene assays yielded similar amplification efficiencies. This method determines the ratio of telomere repeat copy number to single copy gene number in experimental samples relative to a control sample DNA (the relative T/S ratio). The T/S ratio is an arbitary count, but reflects the quanitity of telomeric DNA in relation to the quantity of a single copy DNA sequence. It is an effective measure of the average telomere length [38]. The mean intra-plate coefficient of variation for the telomere and 36B4 assays was 0.56% and 0.19% respectively.

### Measurement of SES

Six SES measures were recorded at wave 5, contemporaneous with telomere measurement: social class based on the household’s current (or most recent) highest ranking occupation [39]; home tenure (home owner, private renter or social renter); income (monthly net household income, equivalised for household size, categorised into quintiles or used as a continuous measure of British Pounds (£) per week); area deprivation based on the Scottish population (Carstairs Index for local authorities, a combination of four indicators from 2001 Census, employed as a continuous variable and as seven category version) [40]–[41]; employment status (employed, caring for the home, retired, unemployed, unable to work due to ill health or other (full-time education, short-term sick, government training scheme, maternity leave)); and subjective social status ladder (respondents’ rating of their own social position, ranging from 1 to 10, relative to others in Britain (McArthur ladder)) [42].

The four measures of childhood SES investigated were: parental social class at age 15 (as above, but based on father’s occupation where available), asked at wave 1; family financial difficulties in childhood up to age 15 (five point scale ranging from ‘very well off’ to ‘often very short of money’); family car ownership in childhood up to age 15 (yes/no); and childhood SES ladder (1–10 scale of subjective assessment of family’s social standing in relation to others in Britain at age 15) asked at wave 5. Data on financial circumstances and family car ownership were collected at wave 3 (and wave 5 if not asked at wave 3).

The two measures of education were collected at wave 5, and so represented the most recent achieved education status. First, years of education (measured continuously and as a binary variable: ≤ or >10 years). Second, educational attainment (none, basic or advanced) was also measured. The specific qualifications included in each category are listed in the footnote to Table 1.

**Table 1:** Characteristics of Study Members, Including Mean Telomere Lengths*.

Cohort	1970s		1950s		1930s	
	Sample size (%of total sample available)	MeanTelomereLength (SE)	Sample size (%)	MeanTelomereLength (SE)	Sample size (%)	MeanTelomereLength (SE)
**Total**	**775 (35.5)**	0.860 (0.008)	**866 (39.6**)	0.784 (0.006)	**544 (24.9)**	0.697 (0.008)
**Sex**						
Male	**362 (46.7**)	0.853 (0.012)	**397 (45.8)**	0.773 (0.009)	**235 (43.2)**	0.676 (0.012)
Female	**413 (53.3)**	0.866 (0.010)	**469 (54.2)**	0.794 (0.009)	**309 (56.8)**	0.714 (0.010)
**Contemporaneous SES measures**						
**Social class**						
I (Professional etc occupations)	**102 (13.2**)	0.871 (0.021)	**123 (14.2)**	0.793 (0.017)	**42 (7.7)**	0.708 (0.029)
II (Managerial and technical occupations)	**396 (51.1)**	0.856 (0.011)	**348 (40.1)**	0.795 (0.010)	**150 (27.6)**	0.690 (0.015)
III – NM (Non-manual skilled occupations)	**151 (19.5)**	0.851 (0.017)	**178 (20.6)**	0.772 (0.014)	**128 (23.5)**	0.726 (0.017)
III – M (Manual skilled occupations)	**74 (9.6)**	0.890 (0.024)	**116 (13.4)**	0.784 (0.017)	**110 (20.2)**	0.681 (0.018)
IV (Partly-skilled occupations)	**46 (5.9)**	0.842 (0.031)	**77 (8.9)**	0.767 (0.021)	**76 (14.0)**	0.682 (0.021)
V (Unskilled occupations)	**5 (0.6)**	0.944 (0.094)	**24 (2.8)**	0.749 (0.038)	**38 (7.0)**	0.698 (0.030)
Missing	**1 (0.1)**		**0 (0)**		**0 (0)**	
**Home tenure**						
Owner	**618 (79.7)**	0.871 (0.009)	**710 (82.0)**	0.783 (0.007)	**404 (74.3)**	0.692 (0.009)
Renter (Private)	**55 (7.1)**	0.861 (0.026)	**27 (3.1)**	0.733 (0.035)	**19 (3.5)**	0.694 (0.033)
Renter (Social)	**96 (12.4)**	0.820 (0.018)	**127 (14.7)**	0.740 (0.014)	**120 (22.0)**	0.666 (0.014)
Missing	**6 (0.8)**		**2 (0.2)**		**1 (0.2)**	
**Income (quintiles)**						
1– Highest	**149 (19.2)**	0.856 (0.018)	**157 (18.1)**	0.780 (0.015)	**85 (15.6)**	0.681 (0.022)
2	**150 (19.4)**	0.827 (0.017)	**159 (18.4)**	0.790 (0.016)	**85 (15.6)**	0.728 (0.023)
3	**150 (19.4)**	0.904 (0.017)	**157 (18.1)**	0.774 (0.014)	**87 (16.0)**	0.705 (0.019)
4	**149 (19.2)**	0.870 (0.015)	**158 (18.2)**	0.771 (0.015)	**83 (15.3)**	0.677 (0.020)
5– Lowest	**150 (19.4)**	0.845 (0.18)	**157 (18.1)**	0.811 (0.015)	**85 (15.6)**	0.713 (0.019)
Missing	**27 (3.4)**		**78 (9.1)**		**119 (21.9)**	
**Area-based deprivation** ¥						
1– Most affluent	**16 (2.1)**	0.877 (0.053)	**34 (3.9)**	0.859 (0.032)	**15 (2.8)**	0.776 (0.048)
2	**125 (16.1)**	0.869 (0.019)	**140 (16.2)**	0.786 (0.016)	**75 (13.8)**	0.697 (0.022)
3	**99 (12.8)**	0.857 (0.021)	**130 (15.0)**	0.767 (0.016)	**73 (13.4)**	0.715 (0.022)
4	**169 (21.8)**	0.864 (0.016)	**202 (23.3)**	0.800 (0.013)	**127 (23.3)**	0.688 (0.017)
5	**95 (12.3)**	0.845 (0.022)	**137 (15.8)**	0.759 (0.016)	**77 (14.2)**	0.706 (0.021)
6	**110 (14.2)**	0.863 (0.020)	**82 (9.5)**	0.767 (0.021)	**77 (14.2)**	0.695 (0.021)
7 - Least affluent	**87 (11.2)**	0.831 (0.023)	**115 (13.3)**	0.796 (0.017)	**92 (16.9)**	0.688 (0.019)
Missing	**74 (9.5)**		**26 (3.0)**		**8 (1.5)**	
**Employment status**						
Employed	**632 (81.5)**	0.866 (0.008)	**651 (75.2)**	0.789 (0.007)	**12 (2.2)**	0.657 (0.042)
Caring for the home	**45 (5.8)**	0.847 (0.033)	**40 (4.6)**	0.821 (0.036)	**16 (2.9)**	0.647 (0.043)
Retired	**0 (0)**	–	**55 (6.4)**	0.760 (0.024)	**506 (93.0)**	0.701 (0.008)
Unemployed	**23 (3.0)**	0.865 (0.047)	**23 (2.7)**	0.780 (0.041)	**0 ((0)**	–
Unable to work via ill health	**26 (3.4)**	0.800 (0.041)	**74 (8.5)**	0.756 (0.020)	**9 (1.7)**	0.669 (0.046)
Other†	**46 (5.9)**	0.808 (0.025)	**21 (2.4)**	0.747 (0.027)	**0 (0)**	–
Missing	**3 (0.4)**		**2 (0.2)**		**1 (0.2)**	
**SES Ladder**						
10 (highest)	**2 (0.3)**	0.969 (0.149)	**6 (0.7)**	0.764 (0.076)	**11 (2.0)**	0.712 (0.056)
9	**19 (2.5)**	0.930 (0.048)	**30 (3.5)**	0.799 (0.034)	**14 (2.6)**	0.739 (0.049)
8	**112 (14.5)**	0.848 (0.020)	**139 (16.1)**	0.794 (0.016)	**65 (11.9)**	0.679 (0.023)
7	**190 (24.5)**	0.873 (0.015)	**212 (24.5)**	0.792 (0.013)	**99 (18.2)**	0.712 (0.019)
6	**190 (24.5)**	0.868 (0.015)	**191 (22.1)**	0.791 (0.014)	**137 (25.2)**	0.693 (0.016)
5	**106 (13.7)**	0.863 (0.020)	**122 (14.1)**	0.779 (0.017)	**102 (18.8)**	0.695 (0.018)
4	**74 (9.5)**	0.815 (0.024)	**73 (8.4)**	0.766 (0.022)	**52 (9.6)**	0.690 (0.026)
3	**44 (5.7)**	0.827 (0.032)	**50 (5.8)**	0.768 (0.026)	**28 (5.1)**	0.705 (0.035)
2	**16 (2.1)**	0.892 (0.053)	**17 (2.0)**	0.719 (0.045)	**6 (1.1)**	0.716 (0.076)
1 (lowest)	**4 (0.5)**	0.918 (0.105)	**7 (0.8)**	0.784 (0.071)	**3 (0.6)**	0.814 (0.107)
Missing	**18 (2.3)**		**19 (2.2)**		**27 (5.0)**	
**Education measures**						
**Education (years)**						
>10 years	**721 (93.0)**	0.859 (0.008)	**546 (63.0)**	0.791 (0.008)	**169 (31.1)**	0.698 (0.014)
≤10 years	**51 (6.6)**	0.862 (0.029)	**313 (36.1)**	0.773 (0.011)	**353 (64.9)**	0.699 (0.010)
Missing	**3 (0.4)**		**7 (0.8)**		**22 (4.0)**	
**Education (qualifications)** ‡						
Advanced	**204 (26.3)**	0.877 (0.015)	**274 (31.6)**	0.770 (0.015)	**93 (17.1)**	0.683 (0.020)
Basic	**528 (68.1)**	0.855 (0.009)	**397 (45.8)**	0.783 (0.009)	**186 (34.2)**	0.723 (0.014)
None	**36 (4.6)**	0.818 (0.035)	**165 (19.1)**	0.770 (0.011)	**221 (40.6)**	0.681 (0.013)
Missing	**7 (0.9)**		**30 (3.5)**		**44 (8.1)**	
**Childhood SES measures**						
**Parental social class at 15**						
I	**74 (9.5)**	0.843 (0.024)	**40 (4.6)**	0.825 (0.030)	**23 (4.2)**	0.743 (0.038)
II	**166 (21.4)**	0.890 (0.016)	**164 (18.9)**	0.805 (0.015)	**86 (15.8)**	0.679 (0.020)
III – NM	**103 (13.3)**	0.869 (0.021)	**79 (9.1)**	0.776 (0.021)	**38 (7.0)**	0.760 (0.030)
III – M	**265 (34.2)**	0.849 (0.013)	**308 (35.6)**	0.780 (0.011)	**310 (57.0)**	0.699 (0.010)
IV	**108 (13.9)**	0.869 (0.020)	**149 (17.2)**	0.775 (0.015)	**32 (5.9)**	0.639 (0.033)
V	**47 (6.1)**	0.805 (0.031)	**89 (10.3)**	0.780 (0.020)	**23 (4.2)**	0.709 (0.038)
Missing	**12 (1.5)**		**57 (6.6)**		**32 (5.9)**	
**Household financial circumstances in childhood**						
Very well off	**12 (1.5)**	1.034 (0.060)	**6 (0.7)**	0.805 (0.077)	**7 (1.3)**	0.802 (0.071)
Quite well off	**247 (31.9)**	0.877 (0.013)	**151 (17.4)**	0.784 (0.015)	**74 (13.6)**	0.690 (0.022)
Usually had just enough money	**330 (42.6)**	0.838 (0.011)	**374 (43.2)**	0.786 (0.010)	**241 (44.3)**	0.702 (0.012)
Sometimes short of money	**133 (17.2)**	0.862 (0.018)	**183 (21.1)**	0.778 (0.014)	**109 (20.0)**	0.708 (0.018)
Often short of money	**50 (6.5)**	0.856 (0.030)	**67 (7.7)**	0.802 (0.023)	**53 (9.7)**	0.694 (0.026)
Missing	**3 (0.4)**		**85 (9.8)**		**60 (11.0)**	
**Family car ownership in childhood**						
Yes	**552 (71.2)**	0.869 (0.009)	**346 (40.0)**	0.780 (0.010)	**78 (14.3)**	0.693 (0.021)
No	**221 (28.5)**	0.835 (0.014)	**437 (50.5)**	0.789 (0.009)	**407 (74.8)**	0.705 (0.009)
Missing	**2 (0.3)**		**83 (9.6)**		**59 (10.8)**	
**SES Ladder**						
10 (high social position)	**4 (0.5)**	0.918 (0.105)	**4 (0.5)**	0.937 (0.093)	**12 (2.2)**	0.758 (0.053)
9	**5 (0.6)**	0.742 (0.094)	**13 (1.5)**	0.823 (0.052)	**15 (2.8)**	0.720 (0.048)
8	**82 (10.6)**	0.868 (0.023)	**57 (6.6)**	0.772 (0.025)	**52 (9.6)**	0.722 (0.026)
7	**118 (15.2)**	0.873 (0.019)	**101 (11.7)**	0.805 (0.019)	**46 (8.5)**	0.670 (0.027)
6	**136 (17.5)**	0.887 (0.018)	**144 (16.6)**	0.789 (0.016)	**87 (16.0)**	0.685 (0.020)
5	**146 (18.8)**	0.847 (0.017)	**156 (18.0)**	0.777 (0.015)	**97 (17.8)**	0.682 (0.019)
4	**103 (13.3)**	0.844 (0.021)	**165 (19.1)**	0.789 (0.015)	**99 (18.2)**	0.717 (0.019)
3	**110 (14.2)**	0.850 (0.020)	**135 (15.6)**	0.768 (0.016)	**70 (12.9)**	0.691 (0.022)
2	**44 (5.7)**	0.868 (0.032)	**60 (6.9)**	0.779 (0.024)	**39 (7.2)**	0.702 (0.030)
1 (low social position)	**11 (1.4)**	0.811 (0.064)	**13 (1.5)**	0.797 (0.052)	**6 (1.1)**	0.618 (0.075)
Missing	**16 (2.1)**		**18 (2.1)**		**21 (3.9)**	
**SES mobility measures**						
**Social class mobility**						
Stable non-manual	**437 (56.4)**	0.866 (0.010)	**534 (61.7)**	0.793 (0.008)	**304 (55.9)**	0.707 (0.011)
Upwards	**206 (26.6)**	0.838 (0.015)	**111 (12.8)**	0.768 (0.018)	**16 (2.9)**	0.703 (0.047)
Downwards	**58 (7.5)**	0.923 (0.028)	**64 (7.4)**	0.790 (0.023)	**53 (9.7)**	0.723 (0.026)
Stable manual	**65 (8.4)**	0.833 (0.026)	**150 (17.3)**	0.773 (0.015)	**171 (31.4)**	0.673 (0.014)
Missing	**9 (1.2)**		**7 (0.8)**		**0 (0)**	
**Home tenure mobility**						
Stable owner	**323 (41.7)**	0.889 (0.012)	**538 (62.1)**	0.795 (0.008)	**268 (49.3)**	0.691 (0.011)
Upwards	**287 (37.0)**	0.834 (0.012)	**170 (19.6)**	0.781 (0.014)	**136 (25.0)**	0.737 (0.016)
Downwards	**38 (4.9)**	0.846 (0.034)	**36 (4.2)**	0.718 (0.031)	**13 (2.4)**	0.706 (0.052)
Stable renter	**109 (14.1)**	0.845 (0.020)	**117 (13.5)**	0.765 (0.017)	**126 (23.2)**	0.669 (0.017)
Missing	**18 (2.3)**		**5 (0.6)**		**1 (0.2)**	
**Accumulated SES measures**						
**Number of waves in non-manual class**						
5	**205 (26.5)**	0.865 (0.015)	**391 (45.2)**	0.794 (0.010)	**264 (48.5)**	0.710 (0.012)
4	**128 (16.5)**	0.884 (0.019)	**77 (8.9)**	0.804 (0.021)	**20 (3.7)**	0.683 (0.042)
3	**69 (8.9)**	0.823 (0.026)	**45 (5.2)**	0.766 (0.028)	**13 (2.4)**	0.745 (0.052)
2	**49 (6.3)**	0.860 (0.030)	**38 (4.4)**	0.808 (0.031)	**14 (2.6)**	0.711 (0.050)
1	**27 (3.5)**	0.838 (0.041)	**41 (4.7)**	0.761 (0.029)	**29 (5.3)**	0.717 (0.035)
0	**17 (2.2)**	0.796 (0.052)	**82 (9.5)**	0.791 (0.021)	**126 (23.2)**	0.687 (0.017)
Missing	**280 (36.1)**		**192 (22.2)**		**78 (14.3)**	
**Number of waves as home owner**						
5	**164 (21.2)**	0.859 (0.016)	**414 (47.8)**	0.801 (0.009)	**232 (42.6)**	0.700 (0.012)
4	**94 (12.1)**	0.864 (0.022)	**76 (8.8)**	0.764 (0.022)	**63 (11.6)**	0.745 (0.024)
3	**84 (10.8)**	0.852 (0.023)	**48 (5.5)**	0.766 (0.027)	**32 (5.9)**	0.740 (0.033)
2	**74 (9.5)**	0.827 (0.024)	**34 (3.9)**	0.789 (0.032)	**28 (5.1)**	0.752 (0.035)
1	**32 (4.1)**	0.851 (0.037)	**27 (3.1)**	0.762 (0.036)	**22 (4.0)**	0.685 (0.040)
0	**33 (4.3)**	0.864 (0.036)	**69 (8.0)**	0.786 (0.023)	**84 (15.4)**	0.670 (0.020)
Missing	**294 (37.9)**		**198 (22.9)**		**83 (15.3)**	

* All telomere length means reported are unadjusted.

¥ The high number of missing values reported here is due to the relocation of respondents to non-Scottish locations.

† Other includes: full-time education, short-term sick, government training scheme, maternity leave or other description not included here.

‡ Advanced  =  University degree, Level 5 vocational qualification, nursing qualification, teaching qualification, or equivalent.

Basic  =  O-Levels, GCSEs, Standard Grades, A-Levels, Highers, HNC/HND, recognized trade apprenticeship, or equivalent.

None  =  none of the above.

Four measures of SES over time were analysed against telomere length. These included social class mobility between waves 1 and 5 (stable non-manual, upward (i.e. manual to non-manual), downward or stable manual); and home tenure mobility between waves 1 and 5 (stable owner, upward i.e. renter to owner, downward mobile or stable renter). For the 1970s cohort, parental social class and home tenure were used at wave 1, so this is a measure of intergenerational mobility. For the other two cohorts it represents their own mobility in middle and older ages. Accumulated social class (number of waves where a respondent was categorised as non-manual); and accumulated home tenure (number of waves where a respondent was classed as an owner) were also measured. For the two accumulation measures, only those respondents who took part in all five waves were included.

### Statistical Analyses

For descriptive purposes, unadjusted telomere lengths were examined for each SES measure and according to gender, separately for each cohort (Table 1). The three cohorts and both genders were included in a combined Analysis of Variance (ANOVA) test to compare telomeres lengths between them. The three cohorts were then analyzed separately using General Linear Models (GLM) to explore the association between SES and telomere length. Each SES variable was modelled independently controlling for gender and assay variation (by including a fixed effect of assay plate). A fixed effect was employed for the assay plate as SES was correlated with plate in our sample; hence it violates the random effects assumption of non-correlation between the factor of interest (SES) and the ‘random’ factor (plate). Interaction terms between sex and SES were tested and sub-sample analyses were performed where *P*≤0.05 (Table S4). Due to the small numerical values telomere lengths were multiplied by 10 to increase the resolution of the coefficients (except for the study characteristics in Table 1). To ease interpretation of results all SES variables were scored such that a higher score represents greater socioeconomic disadvantage. Thus, a negative coefficient denotes lower SES is associated with shorter telomeres. To illustrate our findings we used the difference in mean relative T/S ratio between the cohorts (adjusted for sex and plate) as a crude estimate of the telomere length attrition expected per year of chronological age. Given this, a coefficient of 0.04 is equivalent to a one year difference in biological age as measured by telomere length. All analyses were weighted to the living baseline sample at wave 5 using inverse probability weights to correct for bias due to drop out [43]. Results were computed using SPSS ver.15 (SPSS Inc, Illinois, USA), employing the ‘Complex Samples’ procedure required when using probability weights.

Although the number of SES variables used in this study is a strength, it is important to consider the effects of multiple comparisons as a possible limitation. For contemporaneous SES, education/childhood SES and SES over time there were eight, seven and four variables, respectively, for each cohort. Given this number of tests, Bonferroni-adjusted significance value thresholds for the contemporaneous SES, childhood SES/education and cumulative SES measures (rather than *P*≤0.05) would be 0.007, 0.006 and 0.013, respectively (Table S8).

## Results

The characteristics of study members and their mean telomere lengths (relative T/S ratio) are presented in Table 1. Telomere lengths were shorter with the increasing ages of the cohorts (ANOVA, *P*<0.001, controlling for sex and plate). A sex difference was present, with women having longer telomeres than men, which was statistically significant in the 1950s and 1930s cohorts (ANOVA, *P* = 0.042 and *P*<0.001, respectively). This was equivalent to women being 5 and 11 years biologically younger than men of the same chronological age, respectively. In the 1970s cohort women also had longer telomeres than men (equivalent to 3 years), but the association was not as strong (*P* = 0.115).

### Contemporaneous SES

In the 1970s cohort (35-year-olds) there were significant, positive associations between contemporaneous SES and telomere length (longer telomeres with higher SES) for home tenure (*P*_trend_ = 0.046) and continuous area deprivation (*P*_trend_ = 0.019) (Table 2). Social renters had a biological age 9.6 years older compared to people of the same chronological age who owned their own home. There was some suggestion of an association between subjective social status and telomere length (*P*_trend_ = 0.109). However, there was a significant sex*SES interaction (*P*<0.001), with a stronger linear trend for shorter telomeres with lower self-assessed status in women (*P*_trend_ = 0.040) compared to men (*P*_trend_ = 0.504) (see Table S4). There was no association between telomere length and social class (*P*_trend_ = 0.980). There was evidence that income was weakly associated with telomere length when analysed as quintiles (*P*_trend_ = 0.065), but not when used as a continuous measure (*P*_trend_ = 0.199). This might suggest a non-linear association. Sub-sample analysis (see Table S5) following an interaction between employment status and sex (*P*<0.001) revealed that in men, those caring for the home had longer telomeres than those in employment (*P*<0.001 for men). However, women caring for the home had shorter telomeres than their employed peers (*P* = 0.021). There was a weak association for unemployed men to have shorter telomeres compared to those employed (*P* = 0.064), but this was not replicated for women. Women unable to work through ill health had shorter telomeres than their employed peers (*P* = 0.003), although this was not replicated in their male counterparts. The numbers of respondents unable to work through ill health, unemployed or caring for the home were very low, meaning these results should be treated with caution.

**Table 2:** Estimated Difference in Telomere Length* Associated With Contemporaneous SES Measures†.

COHORT	1970s					1950s					1930s				
	B‡	SE	*P*	*P_overall_*	*P*_trend_	B‡	SE	*P*	*P_overall_*	*P*_trend_	B‡	SE	*P*	*P_overall_*	*P*_trend_
**Social Class**															
I	**0 (ref)**					**0 (ref)**					**0 (ref)**				
II	**−0.211**	0.235	0.370			**−0.131**	0.174	0.451			**−0.358**	0.261	0.170		
III – NM	**−0.036**	0.265	0.893			**−0.009**	0.187	0.960			**0.086**	0.264	0.744		
III – M	**−0.081**	0.283	0.775			**−0.066**	0.218	0.761			**−0.277**	0.267	0.299		
IV	**−0.225**	0.349	0.519			**0.189**	0.259	0.465			**−0.132**	0.278	0.636		
V	**−0.048**	0.749	0.949	0.905	0.980	**−0.671**	0.312	0.032	0.211	0.922	**−0.037**	0.348	0.915	0.221	0.662
**Home Tenure**															
Owner	**0 (ref)**					**0 (ref)**					**0 (ref)**				
Renter (Private)	**−0.049**	0.233	0.835			**−0.234**	0.267	0.380			**−0.147**	0.306	0.631		
Renter (Social)	**−0.386**	0.186	0.038	0.116	0.046	**−0.031**	0.160	0.846	0.678	0.774	**−0.142**	0.165	0.388	0.663	0.380
**Income (equivalised** **quintiles)**															
1– Highest	**0 (ref)**					**0 (ref)**					**0 (ref)**				
2	**0.242**	0.211	0.252			**−0.279**	0.187	0.136			**−0.319**	0.237	0.179		
3	**0.422**	0.213	0.048			**−0.261**	0.175	0.136			**−0.051**	0.228	0.825		
4	**−0.079**	0.225	0.726			**−0.014**	0.175	0.935			**0.133**	0.242	0.582		
5– Lowest	**−0.246**	0.218	0.259	0.015	0.065	**0.064**	0.179	0.721	0.188	0.274	**−0.176**	0.230	0.445	0.716	0.884
**Income** **(£ per week)**	**0.0004**	0.0004	0.199			**0.0001**	0.0003	0.690			**−0.0001**	0.001	0.906		
**Area-** **deprivation**															
1– Most affluent	**0 (ref)**					**0 (ref)**					**0 (ref)**				
2	**−0.021**	0.441	0.962			**−0.327**	0.330	0.321			**0.014**	0.412	0.972		
3	**0.005**	0.430	0.991			**−0.172**	0.350	0.623			**−0.218**	0.407	0.592		
4	**−0.130**	0.413	0.754			**−0.135**	0.328	0.681			**0.008**	0.392	0.985		
5	**−0.242**	0.409	0.555			**−0.319**	0.348	0.361			**−0.271**	0.411	0.510		
6	**−0.231**	0.435	0.596			**−0.378**	0.351	0.281			**0.104**	0.391	0.791		
7 - Least affluent	**−0.460**	0.434	0.290	0.594	0.052	**−0.037**	0.351	0.916	0.592	0.723	**−0.242**	0.395	0.541	0.501	0.538
**Area-deprivation** **(Carstairs)**	**−0.042**	0.018	0.019			**0.002**	0.014	0.907			**−0.011**	0.016	0.465		
**Employment status** §															
Employed	**0 (ref)**					**0 (ref)**									
Caring for the home	**−0.515**	0.276	0.062			**0.093**	0.314	0.768							
Retired						**−0.373**	0.198	0.060							
Unemployed	**−0.270**	0.408	0.508			**−0.142**	0.393	0.718							
Unable to work through ill health	**−1.040**	0.344	0.003			**−0.181**	0.182	0.319							
Other	**−0.410**	0.294	0.164	0.011		**−0.548**	0.286	0.056	0.167						
**SES Ladder**															
10 (highest)	**0 (ref)**					**0 (ref)**					**0 (ref)**				
9	**−0.191**	1.190	0.108			**−0.287**	0.425	0.500			**0.501**	0.613	0.414		
8	**−1.811**	1.111	0.104			**−0.539**	0.309	0.082			**−0.506**	0.487	0.300		
7	**−1.559**	1.091	0.153			**−0.519**	0.296	0.080			**−0.441**	0.460	0.338		
6	**−1.665**	1.095	0.129			**−0.375**	0.296	0.205			**−0.385**	0.463	0.406		
5	**−2.075**	1.115	0.063			**−0.226**	0.318	0.477			**−0.336**	0.458	0.464		
4	**−1.917**	1.150	0.096			**−0.813**	0.329	0.014			**−0.195**	0.493	0.693		
3	**−2.180**	1.122	0.052			**−0.522**	0.355	0.142			**−0.339**	0.491	0.490		
2	**−1.477**	1.181	0.212			**−1.033**	0.389	0.008			**−0.152**	0.673	0.821		
1 (lowest)	**−2.384**	1.365	0.081	0.246	0.109	**−0.073**	0.861	0.933	0.093	0.371	**0.233**	0.865	0.787	0.684	0.908

* Telomere length measured as relative T/S ratio multiplied by 10.

† Analysis samples are weighted to members of the baseline sample who were still alive at wave 5 and all analyses adjusted for gender, and plate.

‡ Unstandardized regression coefficient.

¶ Interaction (p<0.05) between SES variable and gender identified.

§ Employment was not analyzed for the 1930s cohort as 93% were already retired.

In the 1950s cohort (55-year-olds) there was no statistically significant associations between social class, home tenure, income or area deprivation with telomere length (*P*>0.10). Those in the 2^nd^ and 3^rd^ highest income quintiles had shorter telomeres than those in the highest quintile (Table 2), but this pattern was not continued in the 4^th^ and 5^th^ quintiles. There was some suggestion of differences between subjective social status groups (*P* = 0.093). For employment status, there was a weak association for those already retired to have shorter telomeres than those still in employment (*P* = 0.060). The same pattern was seen between those grouped as ‘other’ and those employed (*P* = 0.056). However, there was a significant sex*SES interaction (*P* = 0.015). Stratified analysis by sex revealed that men unable to work through ill health actually had longer telomeres than their employed peers (*P* = 0.005), while those grouped as ‘other’ had shorter telomeres (*P* = 0.002). For women, those who had already retired had shorter telomeres than their employed peers (*P* = 0.026), with those unable to work through ill health also nearing significance for shorter telomeres (*P* = 0.068) (Table S6).

In the 1930s cohort (75-year-olds), none of the contemporaneous SES variables were significantly associated with telomere length (Table 2), although there was a statistically significant SES*sex interaction (*P* = 0.007) for subjective social status. For men there was no evidence of an association with telomere length, but there was weak evidence for longer telomeres with decreasing social status in women (*P* = 0.065) (Table S7).

### Childhood SES and Education

In the 1970s cohort there was a strong positive association between telomere length and parental social class at 15 (*P*_trend_ = 0.003). The difference between the highest and lowest categories was equivalent to almost 20 biological years for people of the same chronological age. A greater number of years of education (continuous measure) was significantly associated with longer telomere length (*P*_trend_ = 0.003), although not when dichotomised (*P* = 0.409) (Table 3). Sub-sample analysis revealed continuous years of education were positively associated with telomere length for women (*P*_trend_ = 0.001), but not men (*P*_trend_ = 0.698) (see Table S5). Educational achievement was positively associated with telomere length (*P*_trend_ = 0.027), as was family car ownership in childhood (*P* = 0.027). Those in the lowest SES categories for these measures had a biological age 8.5 years older compared to people of the same chronological age in the highest SES group for car ownership and 17 years for education. Although individual categories reporting varying degrees of childhood financial difficulties were not statistically different to those who reported being well off, there was a weak gradient between them as the reported difficulties became more severe (*P*_trend_ = 0.074). There was no association for the retrospective subjective assessment of the family’s social standing at age 15 (*P*_trend_ = 0.423).

**Table 3:** Estimated Difference in Telomere Length* Associated With Education and Childhood SES Measures†.

COHORT	1970s					1950s					1930s				
	B‡	SE	*P*	*P_overall_*	*P*_trend_	B‡	SE	*P*	*P_overall_*	*P*_trend_	B‡	SE	*P*	*P_overall_*	*P*_trend_
**Education (years)**															
>10 years	**0 (ref)**					**0 (ref)**					**0 (ref)**				
≤10 years	**−0.210**	0.254	0.409			**−0.028**	0.118	0.815			**0.255**	0.144	0.078		
															
**Education (years –** **continuous)**	**0.064**	0.022	0.003			**−0.008**	0.018	0.668			**−0.033**	0.025	0.178		
**Education (qualifications)**															
Advanced	**0 (ref)**					**0 (ref)**					**0 (ref)**				
Basic	**−0.279**	0.171	0.104			**0.048**	0.131	0.717			**0.315**	0.200	0.116		
None	**−0.697**	0.334	0.038	0.080	0.027	**−0.054**	0.164	0.741	0.762	0.779	**0.070**	0.191	0.714	0.201	0.796
**Parental class at 15** **(male-dominated)**															
I	**0 (ref)**					**0 (ref)**					**0 (ref)**				
II	**−0.188**	0.317	0.554			**−0.240**	0.298	0.420			**−0.656**	0.4320	0.041		
III-NM	**0.309**	0.334	0.354			**−0.161**	0.334	0.629			**0.382**	0.353	0.280		
III-M	**−0.414**	0.301	0.169			**−0.038**	0.285	0.893			**−0.230**	0.291	0.430		
IV	**−0.404**	0.337	0.231			**−0.247**	0.301	0.412			**−0.444**	0.391	0.257		
V	**−0.772**	0.363	0.034	0.007	0.007	**−0.153**	0.326	0.640	0.728	0.949	**−0.286**	0.393	0.467	0.008	0.856
**Household financial** **difficulties at 15**															
Very well off	**0 (ref)**					**0 (ref)**					**0 (ref)**				
Quite well off	**−0.668**	0.747	0.371			**−0.704**	1.052	0.504			**−0.145**	0.502	0.773		
Usually had just enough money	**−0.956**	0.747	0.201			**−0.624**	1.045	0.550			**−0.101**	0.486	0.835		
Sometimes short of money	**−0.959**	0.766	0.211			**−0.522**	1.051	0.619			**−0.013**	0.486	0.979		
Often short of money	**−1.021**	0.817	0.212	0.279	0.074	**−0.837**	1.057	0.429	0.528	0.835	**−0.113**	0.490	0.818	0.971	0.758
**Family car ownership at 15**															
Yes	**0 (ref)**					**0 (ref)**					**0 (ref)**				
No	**−0.341**	0.153	0.027			**0.266**	0.114	0.020			**0.237**	0.191	0.215		
**SES Ladder**															
10 (highest)	**0 (ref)**					**0 (ref)**					**0 (ref)**				
9	**−0.816**	1.218	0.503			**0.029**	0.647	0.964			**−1.149**	0.615	0.062		
8	**0.245**	1.060	0.817			**−0.517**	0.507	0.308			**−0.353**	0.563	0.531		
7	**0.248**	1.061	0.815			**−0.683**	0.499	0.171			**−1.449**	0.579	0.013		
6	**0.267**	1.072	0.803			**−0.655**	0.492	0.184			**−0.928**	0.533	0.082		
5	**−0.003**	1.052	0.998			**−0.773**	0.489	0.114			**−1.077**	0.541	0.047		
4	**−0.044**	1.064	0.967			**−0.548**	0.491	0.265			**−0.569**	0.554	0.305		
3	**0.112**	1.062	0.916			**−0.648**	0.512	0.207			**−0.963**	0.549	0.080		
2	**−0.119**	1.094	0.913			**−1.089**	0.518	0.036			**−0.718**	0.537	0.182		
1 (lowest)	**0.773**	1.309	0.555	0.588	0.423	**−0.883**	0.601	0.142	0.298	0.095	**−1.694**	0.692	0.015	0.001	0.383

* Telomere length measured as relative T/S ratio multiplied by 10.

† Analysis samples are weighted to members of the baseline sample who were still alive at wave 5 and all analyses adjusted for gender and plate.

‡ Unstandardized regression coefficients.

In the 1950s cohort (Table 3), respondents whose families owned a car during their childhood had longer telomeres than those whose families did not (*P* = 0.020). There was a trend for increasing subjective social status to be positively associated with telomere length (*P*_trend_ = 0.095). A significant SES*sex interaction (*P* = 0.007) was found for financial difficulties at age 15. Sex-stratified analysis revealed that those very well off at age 15 actually had shorter telomeres than those experiencing all other grades of financial difficulties in men (*P*<0.001). There was no association between telomere length and financial difficulties in women (*P* = 0.231). There were no further associations with any of the other childhood SES or education measures.

In the 1930s cohort (Table 3), there were no significant associations between education or childhood SES and telomere length (*P*≤0.05), although there was a weak association between dichotomous years of education and telomere length (*P* = 0.078). However, this association was in the opposite direction to that expected, with less years of education associated with longer telomeres. There was a significant sex*parental class interaction. When the analysis was stratified by sex, it showed there was a strong trend for longer telomeres with higher parental class in men (*P*_trend_ = 0.004), whereas women showed the opposite (*P*_trend_ = 0.028) (Table S6). Although there was a SES*sex interaction for subjective social status at age 15, sub-sample analyses did not reveal a distinct pattern that differed between the two sexes (Table S7).

### SES Over Time

In the 1970s cohort telomere length was positively associated with intergenerational social mobility in home tenure (*P*_trend_ = 0.004), with stable renters having the shortest telomeres, with those who moved from owning to renting having slightly longer telomeres than those who moved from renting to owning and finally stable owners having the longest telomeres (Table 4). Stable renters were approximately 13 years biologically older than same-age stable owners. There were also significant differences between social class mobility groups (*P* = 0.046), although the pattern was not clear. There was no association between the number of waves in the non-manual class or as a home owner with telomere length (*P*_trend_ = 0.348 and *P*_trend_ = 0.663, respectively), although these analyses had a much reduced sample size.

**Table 4:** Estimated Difference in Telomere Length* Associated With Measures of SES Over Time†.

COHORT	1970s					1950s					1930s				
	B‡	SE	*P*	*P_overall_*	*P*_trend_	B‡	SE	*P*	*P_overall_*	*P*_trend_	B‡	SE	*P*	*P_overall_*	*P*_trend_
**Social class mobility**															
Stable non-manual	**0 (ref)**					**0 (ref)**					**0 (ref)**				
Upwards	**−0.390**	0.158	0.014			**−0.008**	0.153	0.958			**−0.344**	0.289	0.235		
Downwards	**0.162**	0.255	0.527			**−0.108**	0.231	0.641			**−0.203**	0.255	0.426		
Stable manual	**−0.280**	0.227	0.218	0.046	0.218	**0.115**	0.155	0.458	0.832	0.581	**−0.036**	0.135	0.793	0.604	0.734
**Home tenure mobility**															
Stable owner	**0 (ref)**					**0 (ref)**					**0 (ref)**				
Upwards	**−0.423**	0.156	0.007			**−0.032**	0.144	0.822			**0.212**	0.171	0.216		
Downwards	**−0.506**	0.277	0.068			**−0.155**	0.287	0.589			**−0.843**	0.323	0.009		
Stable renter	**−0.553**	0.200	0.006	0.010	0.004	**−0.027**	0.167	0.870	0.956	0.765	**−0.006**	0.186	0.976	0.015	0.789
**Number of waves in** **non-manual class**															
5	**0 (ref)**					**0 (ref)**					**0 (ref)**				
4	**0.045**	0.201	0.842			**0.033**	0.214	0.876			**−0.432**	0.307	0.160		
3	**−0.245**	0.225	0.278			**−0.264**	0.247	0.285			**0.117**	0.317	0.713		
2	**0.083**	0.294	0.777			**−0.280**	0.320	0.381			**0.321**	0.298	0.282		
1	**−0.310**	0.381	0.416			**−0.003**	0.200	0.987			**−0.325**	0.338	0.337		
0	**−0.322**	0.351	0.359	0.688	0.348	**0.236**	0.197	0.230	0.572	0.475	**−0.088**	0.164	0.591	0.462	0.562
**Number of waves as** **home owner**															
5	**0 (ref)**					**0 (ref)**					**0 (ref)**				
4	**0.149**	0.244	0.542			**−0.365**	0.176	0.039			**0.545**	0.212	0.011		
3	**−0.143**	0.251	0.570			**−0.293**	0.278	0.292			**0.233**	0.330	0.481		
2	**−0.193**	0.243	0.429			**0.045**	0.287	0.875			**0.500**	0.390	0.201		
1	**−0.048**	0.315	0.880			**−0.619**	0.346	0.074			**−0.122**	0.369	0.741		
0	**0.026**	0.333	0.937	0.838	0.663	**0.100**	0.204	0.625	0.136	0.755	**−0.278**	0.191	0.145	0.034	0.130

* Telomere length measured as relative T/S ratio multiplied by 10.

† Analysis samples are weighted to members of the baseline sample who were still alive at wave 5 and all analyses adjusted for gender and plate.

‡ Unstandardized regression coefficients.

In the 1950s cohort there were no associations between the measures of SES over time and telomere length (Table 4). In the 1930s cohort, there were significant differences between home tenure mobility groups (*P* = 0.015), with those having experienced downward mobility having shorter telomeres than stable owners (*P* = 0.009). There was also evidence of significant differences in the accumulated home ownership analysis (*P* = 0.034).

### Multiple Comparisons

The Bonferroni-adjusted p-values of 0.007 for the contemporaneous SES measures, 0.006 for the education and childhood SES measures and 0.013 for the SES over time measures do alter the number of tests that could be considered statistically significant. In the 1970s cohort, adjusting the significance thresholds resulted in all tests being non-significant for the contemporaneous SES measures. For the childhood SES and education tests, only years of education and parental social class remained significant in the 1970s cohort. In the accumulated SES analysis, home tenure mobility remained statistically significant for the 1970s cohort. For the 1950s and 1930s cohorts, all tests for contemporaneous SES, childhood SES, education and over time would be considered not statistically significant.

## Discussion

The analysis presented here has mixed findings across three age cohorts for associations between telomere length and SES. In the youngest cohort, with respondents aged around 35, in general those in the highest SES groups, whether measured contemporaneously, by education, in childhood or over time, had longer telomeres (although the evidence was for more childhood SES/education measures to be associated with telomere length than contemporaneous or cumulative SES measures). There were few significant associations for the older two cohorts aged approximately 55 and 75.

Previous studies of SES and telomere length have shown mixed results also. Studies including markers of various contemporaneous SES measures have predominantly found null associations [4], [18]–[19], [22]–[26], [29]–[34], while a smaller number have found positive (higher SES and longer telomeres) [12], [31], [34] or negative associations [16]–[17]. For education, null associations [4], [12], [18], [20]–[21], [25], [27]–[28], [33] have outnumbered positive associations [14]–[15], [32], [34], while childhood measures have shown examples of positive [13], as well as null associations [4], [19], [29]. Only one previous study has utilised SES measures over time, with all four measures included showing no association with telomere length [19]. However, many of the studies have been limited by: small sample sizes [13]–[14], [17], [19], [21]–[28], [30], [32]–[34]; being case-control studies [4], [14], [21]–[22], [25], [30]; using non-representative samples [12]–[13], [17], [31], [34]; having age ranges excluding younger respondents [4], [15]–[16], [18]–[20], [23]–[24], [26], [28]–[30], [32], [34]; and/or using limited SES measures (only one SES measure and/or only used as a binary predictor) [12]–[17], [20]–[21], [23]–[24], [26]–[28], [30]–[31], [33]. Across these studies there does not appear to be any discernible pattern in terms of SES-telomere associations linked to the age structure, study design (case-control vs. general population), geographical location, telomere length measurement technique (qPCR vs. southern blot) or SES measure. The differences in findings may reflect weak associations between SES and telomere length, and/or different quality studies. The results of this study add to the already mixed picture.

The sex difference found in the Twenty-07 Study, with women having longer telomeres than men, is consistent with other population-based studies [15]–[16], [18], [28], [32], [44]–[45]. Those studies that found no such differences between men and women typically included younger respondents [12], [33], [46]–[48], with our study finding a weaker association in the younger cohort, but still in the expected direction.

Steptoe et al (2011) hypothesised that early life SES measures would be more strongly associated with telomere length than contemporaneous SES measures as a result of sensitive period effects [34]. The evidence here does show that in the 1970s cohort, at least, education and childhood SES measures were more readily associated with telomere length compared to contemporaneous measures of SES (four out of seven significant results compared to three out of eight, respectively). However, across the literature, markers of education or childhood SES have not been found to be any more readily associated with telomere length compared to contemporaneous SES.

The strongest theoretical pathway between SES and telomere length is via oxidative stress, which is the imbalance of DNA- (and therefore telomere) damaging compounds (oxidants) over the protective compounds (antioxidants) [49]–[50]. Oxidative stress levels are increased by physical and mental stress, as well as poor nutrition and unhealthy behaviours [50], all of which increase with disadvantaged SES [2]. Given this, it has been suggested that long-term exposure to lower SES should result in greater telomere attrition as there has been longer exposure to potentially damaging environments such as lower SES [19], [34]. We find only minimal support for this with one out of four cumulative SES measures being significant. However, the analyses involving these accumulated measures had much-reduced sample sizes owing to the fact that respondents had to take part in all five waves, and so this may reflect a lack of statistical power. Other studies have less extensive SES measures with which to consider the different key life stages and lifecourse models in the association between SES and telomere length, with only Adams et al (2007) also having measures over time [19]. Although it must be noted that Adams et al found no statistically significant associations between cumulative SES and telomere length.

As cellular damage accumulates with age, including reductions in telomere length, the SES-telomere association seen in younger individuals may become diluted as other factors such as disease and psychosocial factors have a greater influence on the rate of telomere loss. However, disease and psychosocial factors are socially-patterned, meaning that differences between high and low SES groups would more likely be maintained or even increase over time, rather than decrease. However, we do not find this here, which may be the result of survival bias. A greater number of deaths had occurred in the two older cohorts compared to the youngest cohort before telomere length was measured, especially those with lower SES and poorer health (Tables S1, S2, S3). These individuals potentially have shorter telomeres, thereby reducing the observed associations at older ages. Correcting the analyses using weights allows the problems of selective drop-out (higher in lower SES individuals) to be addressed, but cannot correct for survival bias. The survival bias identified in the older cohorts is a strong indicator for the lack of associations. As many of the other studies of telomere length and SES have focused on older cohorts, survival bias could be a major issue throughout the literature that is not being given proper consideration. An alternative explanation to survival bias is that differences in the SES-telomere association by cohort may reflect cohort effects. Other studies have found that the effect of SES on health increases with younger birth cohorts [51]. It has been suggested that this is the result of the changing contexts for the SES-health association. For example, the differences in the meaning of different SES measures have changed for different cohorts (e.g. the growing importance of education in people’s lives with younger birth cohorts); life expectancy has increased with younger cohorts; and the pattern of diseases has also altered across cohorts. While it is not possible in this study to unravel age and cohort effects, the possibility that the differences observed may be due to cohort effects, rather than age, may explain these results, and differences between other studies in the literature.

In this study we have attempted to minimise some of the limitations of previous studies by using a relatively large (for this literature), community-based tri-cohort study, with a wide range of SES measures across key periods of the lifecourse. A potential weakness, however, is the age structure of the Twenty-07 Study, made up of three cohorts, each 20 years apart. This lack of a continuous age range does somewhat limit the conclusions that can be made about the ages not sampled here, although it gives a good indication of the association at key life stages. It also alerts us to differences in the association by age that are masked in single age cohort studies or studies based on a continuous age range. Given the number of SES measures used, it is important to consider the introduction of Type I errors (false positives) introduced by such multiple testing. Bonferroni-adjusted significance values did reduce the number of tests found to be statistically significant in the 1970s cohort, however it should be noted that the Bonferroni is a conservative estimate and assumes that the multiple tests being compared are independent (which is not the case with SES measures). Hence, the Bonferroni (and other similar adjustments) introduce Type II errors (false negatives) that will typically reduce the likelihood of finding statistically significant results.

The effectiveness of telomere length as a measure of biological ageing has been questioned, with results still equivocal [52]–[53]. One of the major issues relating to its use is the possibility that telomere length is an imprecise marker of biological ageing [6], [54]. Telomere length is typically measured in leukocytes, but leukocytes are made up of a heterogeneous mix of cell types of different ages, which may result in a range of telomere lengths [53], [55]. Even within a specific cell type there can be variability in the lengths of telomeres [56]. Currently, it is not possible to resolve these issues with the methods available to measure telomere length. A measure of telomere attrition in individuals (via repeated telomere length measurements) may be required to investigate the association more effectively [57], although precision issues may still mask potential associations [54]. There has also been some evidence that telomere length becomes increasingly ineffective as a measure of ageing in the elderly (70 and above) due to telomeric instability (telomeres having been shown to increase or cease shortening) [24], [58], which may help explain the lack of association in the 1930s cohort who were aged approximately 75. Moreover, given higher levels of morbidity and deprivation than their UK and European counterparts (‘the Glasgow Effect’) [59], it may be possible that 55-year-olds in Glasgow are biologically older than same-age individuals from other cities, driving this telomere instability even at this early age. An additional problem is the effectiveness of the techniques used to measure telomere length. There is some evidence of increased variation in qPCR techniques over southern blot, which may reduce our ability to detect small differences between, for example, high and low SES categories [55], [60]. However, qPCR is more cost-effective than other techniques such as southern blot, especially on a large-scale, and the results of qPCR have been shown to be strongly correlated with other techniques [54].

In this study there appears to be evidence of a relationship between shorter telomeres and poorer levels of contemporaneous and childhood SES, less education and those experiencing intergenerational, downward social mobility, at age 35. However, the strongest evidence appears to be from the education and childhood SES measures, possibly representing the effects of a sensitive period for telomere attrition. Few associations are seen in those aged 55 or 75. This may be the result of a socially-patterned survival bias, cohort differences or because telomere is a less reliable marker of biological ageing at middle and older ages in a population with high levels of early morbidity and mortality.

## Supporting Information

Table S1 Sample sizes for death, drop-out and telomere samples at wave 5.
(DOCX)

Table S2 Odds ratios (OR) for the risk of death by wave 5 given socioeconomic and health characteristics at wave 1.
(DOCX)

Table S3 Odds ratios for the risk of drop-out by wave 5 given socioeconomic and health characteristics at wave 1.
(DOCX)

Table S4 Significance values for the sex interaction term in the main analysis GLMs.
(DOCX)

Table S5 Estimated difference in telomere length associated with employment status, subjective social class/MacArthur ladder and years of education for the 1970s cohort.
(DOCX)

Table S6 Estimated difference in telomere length associated with employment status and household financial difficulties at age 15 for the 1950s cohort.
(DOCX)

Table S7 Estimated difference in telomere length associated with subjective social class/MacArthur ladder at ages 15 and 75, and parental social class at age 15 for the 1930s cohort.
(DOCX)

Table S8 Bonferroni adjusted P-values according to the three key life stages where SES was measured.
(DOCX)

## References

[1] Marmot MG (2010). The Marmot Review: Fair society, Healthy Lives. Strategic review of health inequalities in England post-2010.. London: The Marmot Review.

[2] Adams JM, White M (2004). Biological ageing: a fundamental, biological link between socio-economic status and health?. Eur J Public Health 14.

[3] Adler NE, Stewart J (2010). Health disparities across the lifespan: meaning, methods, and mechanisms.. Ann N Y Acad Sci.

[4] Batty GD, Wang Y, Brouilette SW, Shiels P, Packard C (2009). Socioeconomic status and telomere length: the West of Scotland Coronary Prevention Study.. J Epidemiol Community Health 63.

[5] de Lange T (2002). Protection of mammalian telomeres.. Oncogene 21.

[6] Shiels PG (2010). Improving Precision in Investigating Aging: Why Telomeres Can Cause Problems.. J Gerontol A Biol Sci Med Sci 65.

[7] von Zglinicki T, Serra V, Lorenz M, Saretzki G, Lenzen-Grossimlighaus R (2000). Short telomeres in patients with vascular dementia: An indicator of low antioxidative capacity and a possible risk factor?. Lab Invest 80.

[8] Carrero J, Stenvinkel P, Fellstrom B, Qureshi AR, Lamb K (2008). Telomere attrition is associated with inflammation, low fetuin-A levels and high mortality in prevalent haemodialysis patients.. J Intern Med 263.

[9] Blasco MA (2005). Telomeres and human disease: Ageing, cancer and beyond.. Nat Rev Genet 6.

[10] Maxwell F, McGlynn LM, Muir HC, Talwar D, Benzeval M (2011). Telomere attrition and decreased Fetuin A levels indicate accelerated biological ageing and are implicated in the pathogenesis of Colorectal Cancer.. Clin Cancer Res 17.

[11] Cawthon RM, Smith KR, O’Brien E, Sivatchenko A, Kerber RA (2003). Association between telomere length in blood and mortality in people aged 60 years or older.. Lancet 361.

[12] Cherkas LF, Hunkin JL, Kato BS, Richards JB, Gardner JP (2008). The association between physical activity in leisure time and leukocyte telomere length.. Arch Intern Med 168.

[13] Deroo LA, Parks CG, Kim S, Cawthon RM, Weinberg C (2009). Childhood Socioeconomic Factors and Telomere Length.. Am J Epidemiol.

[14] Hou LF, Savage SA, Blaser MJ, Perez-Perez G, Hoxha M (2009). Telomere Length in Peripheral Leukocyte DNA and Gastric Cancer Risk.. Cancer Epidem Biomar 18.

[15] Yaffe K, Lindquist K, Kluse M, Cawthon R, Harris T (2011). Telomere length and cognitive function in community-dwelling elders: Findings from the Health ABC Study.. Neurobiol Aging 32.

[16] Woo J, Suen EWC, Leung JCS, Tang NLS, Ebrahim S (2009). Older men with higher self-rated socioeconomic status have shorter telomeres.. Age Ageing 38.

[17] Parks CG, DeRoo LA, Miller DB, McCanlies EC, Cawthon RM (2011). Employment and work schedule are related to telomere length in women.. Occup Environ Med 68.

[18] Harris SE, Martin-Ruiz C, von Zglinicki T, Starr JM, Deary IJ (2012). Telomere length and aging biomarkers in 70-year-olds: the Lothian Birth Cohort 1936.. Neurobiol Aging 33.

[19] Adams J, Martin-Ruiz C, Pearce MS, White M, Parker L (2007). No association between socio-economic status and white blood cell telomere length.. Aging Cell 6.

[20] Chan R, Woo J, Suen E, Leung J, Tang N (2010). Chinese tea consumption is associated with longer telomere length in elderly Chinese men.. Br J Nutr 103.

[21] Epel ES, Blackburn EH, Lin J, Dhabhar FS, Adler NE (2004). Accelerated telomere shortening in response to life stress.. Proc Natl Acad Sci U S A 101.

[22] Fernandez-Egea E, Bernardo M, Heaphy CM, Griffith JK, Parellada E (2009). Telomere Length and Pulse Pressure in Newly Diagnosed, Antipsychotic-Naive Patients With Nonaffective Psychosis.. Schizophr Bull 35.

[23] Geronimus AT, Hicken MT, Pearson JA, Seashols SJ, Brown KL (2010). Do US Black Women Experience Stress-Related Accelerated Biological Aging?. Hum Nature-Int Bios 21.

[24] Harris SE, Deary IJ, Maclntyre A, Lamb KJ, Radhakrishnan K (2006). The association between telomere length, physical health, cognitive ageing, and mortality in non-demented older people.. Neurosci Lett 406.

[25] Kananen L, Surakka I, Pirkola S, Suvisaari J, Lonnqvist J (2010). Childhood adversities are associated with shorter telomere length at adult age both in individuals with an anxiety disorder and controls.. PLoS ONE 5.

[26] Mather KA, Jorm AF, Anstey KJ, Milburn PJ, Easteal S (2010). Cognitive performance and leukocyte telomere length in two narrow age-range cohorts: a population study.. BMC Geriatr.

[27] Nordfjall K, Eliasson M, Stegmayr B, Lundin S, Roos G (2008). Increased abdominal obesity, adverse psychosocial factors and shorter telomere length in subjects reporting early ageing; the MONICA Northern Sweden Study.. Scand J Public Health 36.

[28] Risques RA, Arbeev KG, Yashin AI, Ukraintseva SV, Martin GM (2010). Leukocyte Telomere Length Is Associated with Disability in Older US Population.. J Am Geriatr Soc 58.

[29] Surtees PG, Wainwright NW, Pooley KA, Luben RN, Khaw KT (2011). Life Stress, Emotional Health, and Mean Telomere Length in the European Prospective Investigation into Cancer (EPIC)-Norfolk Population Study.. J Gerontol A Biol Sci Med Sci 66.

[30] Zheng YL, Ambrosone C, Byrne C, Davis W, Nesline M (2010). Telomere length in blood cells and breast cancer risk: investigations in two case-control studies.. Breast Cancer Res Treat 120.

[31] Cherkas LF, Aviv A, Valdes AM, Hunkin JL, Gardner JP (2006). The effects of social status on biological aging as measured by white-blood-cell telomere length.. Aging Cell 5.

[32] Diez Roux AV, Ranjit N, Jenny NS, Shea S, Cushman M (2009). Race/ethnicity and telomere length in the Multi-Ethnic Study of Atherosclerosis.. Aging Cell 8.

[33] Shiels PG, McGlynn LM, MacIntyre A, Johnson PCD, Batty GD (2011). Accelerated Telomere Attrition Is Associated with Relative Household Income, Diet and Inflammation in the pSoBid Cohort.. PLoS ONE 6.

[34] Steptoe A, Hamer M, Butcher L, Lin J, Brydon L (2011). Educational attainment but not measures of current socioeconomic circumstances are associated with leukocyte telomere length in healthy older men and women.. Brain Behav Immun 25.

[35] Macintyre S, Annandale E, Ecob R, Ford G, Hunt K, Martin CJ, McQueen DV (1989). The West of Scotland Twenty-07 Study: health in the community..

[36] Benzeval M, Der G, Ellaway A, Hunt K, Sweeting H (2009). Cohort Profile: West of Scotland Twenty-07 Study: Health in the Community.. Int J Epidemiol 38.

[37] Lorimer K, Green M, Shipton D, Benzeval M (2010). The West of Scotland Twenty-07 Study: Health in the Community, Wave 5 Fieldwork Report, 2007/8.. MRC/CSO Social and Public Health Sciences Unit Working Paper 26, Glasgow.

[38] Cawthon RM (2002). Telomere measurement by quantitative PCR.. Nucleic Acids Res 30.

[39] OPCS (1980). Classification of occupations 1980.. London: HMSO.

[40] Carstairs V, Morris R (1991). Deprivation and health in Scotland. Aberdeen: Aberdeen Univ.. Press.

[41] McLoone P (2004). Carstairs scores for Scottish postcode sectors from the 2001 Census.. Glasgow: MRC/CSO Social and Public Health Sciences Unit, Glasgow.

[42] Adler NE, Epel ES, Castellazzo G, Ickovics JR (2000). Relationship of subjective and objective social status with psychological and physiological functioning: Preliminary data in healthy white women.. Health Psychol 19.

[43] Seaman S, Benzeval M (2011). The West of Scotland Twenty-07 Study: Inverse probability weights for Wave 5.. MRC/CSO Social and Public Health Sciences Unit Working Paper 27, Glasgow.

[44] Benetos A, Okuda K, Lajemi M, Kimura M, Thomas F (2001). Telomere length as an indicator of biological aging: the gender effect and relation with pulse pressure and pulse wave velocity.. Hypertension 37.

[45] Nawrot TS, Staessen JA, Gardner JP, Aviv A (2004). Telomere length and possible link to X chromosome.. Lancet 363.

[46] De Meyer T, Rietzschel ER, De Buyzere ML, De Bacquer D, Van Criekinge W (2007). Paternal age at birth is an important determinant of offspring telomere length.. Hum Mol Genet 16.

[47] Lee M, Martin H, Firpo MA, Demerath EW (2011). Inverse association between adiposity and telomere length: The Fels Longitudinal Study.. Am J Hum Biol 23.

[48] Unryn BM, Cook LS, Riabowol KT (2005). Paternal age is positively linked to telomere length of children.. Aging Cell 4.

[49] Janicki-Deverts D, Cohen S, Matthews KA, Gross MD, Jacobs DR (2009). Socioeconomic Status, Antioxidant Micronutrients, and Correlates of Oxidative Damage: The Coronary Artery Risk Development in Young Adults (CARDIA) Study.. Psychosom Med 71.

[50] Sies H (1991). Oxidative stress : oxidants and antioxidants.. London; San Diego: Academic Press.

[51] Lynch SM (2003). Cohort and life-course patterns in the relationship between education and health: a hierarchical approach.. Demography 40.

[52] von Zglinicki T, Martin-Ruiz CM (2005). Telomeres as biomarkers for ageing and age-related diseases.. Curr Mol Med 5.

[53] Mather KA, Jorm AF, Parslow RA, Christensen H (2011). Is telomere length a biomarker of aging? A review.. J Gerontol A Biol Sci Med Sci 66.

[54] Chen W, Kimura M, Kim S, Cao X, Srinivasan SR (2011). Longitudinal versus cross-sectional evaluations of leukocyte telomere length dynamics: age-dependent telomere shortening is the rule.. J Gerontol A Biol Sci Med Sci 66.

[55] Aviv A, Valdes AM, Spector TD (2006). Human telomere biology: pitfalls of moving from the laboratory to epidemiology.. Int J Epidemiol 35.

[56] Aubert G, Lansdorp PM (2008). Telomeres and aging.. Physiol Rev 88.

[57] Kuh D (2006). A life course perspective on telomere length and social inequalities in aging.. Aging Cell 5.

[58] Martin-Ruiz CM, Gussekloo J, van Heemst D, von Zglinicki T, Westendorp RG (2005). Telomere length in white blood cells is not associated with morbidity or mortality in the oldest old: a population-based study.. Aging Cell 4.

[59] Walsh DT M, Hanlon P (2008). The Aftershock of Deindustrialisation: Trends in mortality in Scotland and other parts of post-industrial Europe.. Glasgow: Glasgow Centre for Population Health.

[60] Aviv A, Hunt SC, Lin J, Cao X, Kimura M (2011). Impartial comparative analysis of measurement of leukocyte telomere length/DNA content by Southern blots and qPCR.. Nucleic Acids Res 39.

