# Supplementary material for: Is Telomere Length Socially Patterned? Evidence from the West of Scotland Twenty-07 Study
Source: PLoS One. 2012 Jul 23;7(7):e41805. doi: 10.1371/journal.pone.0041805 (PMC3402400; doi:10.1371/journal.pone.0041805)
Supplement: Table S1 — Sample sizes for death, drop-out and telomere samples at wave 5. (DOCX) [file pone.0041805.s001.docx]

**Table S1** Sample sizes for death, drop-out and telomere samples at wave 5

|  | **1970s** | **1950s** | **1930s** |
| --- | --- | --- | --- |
|  | **N (%)** | **N (%)** | **N (%)** |
| **Dead by wave 5** |  |  |  |
| Yes | 25 (1.7%) | 87 (6.0%) | 562 (36.2%) |
| No | 1490 (98.3%) | 1357 (94.0%) | 989 (63.8%) |
| **Dropped out before wave 5** |  |  |  |
| Yes | 549 (36.2%) | 358 (24.8%) | 326 (21.0%) |
| No | 966 (63.8%) | 1086 (75.2%) | 1225 (79.0%) |
| **Telomere sample available** |  |  |  |
| Yes | 774 (51.1%) | 866 (60.0%) | 544 (35.1%) |
| No | 741 (48.9%) | 578 (40.0%) | 1007 (64.9%) |
|  |  |  |  |
