# Supplementary material for: Is Telomere Length Socially Patterned? Evidence from the West of Scotland Twenty-07 Study
Source: PLoS One. 2012 Jul 23;7(7):e41805. doi: 10.1371/journal.pone.0041805 (PMC3402400; doi:10.1371/journal.pone.0041805)
Supplement: Table S3 — Odds ratios for the risk of drop-out by wave 5 given socioeconomic and health characteristics at wave 1. (DOCX) [file pone.0041805.s003.docx]

**Table S3** Odds ratios for the risk of drop-out by wave 5 given socioeconomic and health characteristics at wave 1

|  | **1970s** | | | | **1950s** | | | | **1930s** | | | |
| --- | --- | --- | --- | --- | --- | --- | --- | --- | --- | --- | --- | --- |
|  | **Odds Ratio** | **95% CI (low)** | **95% CI (high)** | ***P*** | **Odds Ratio** | **95% CI (low)** | **95% CI (high)** | ***P*** | **Odds Ratio** | **95% CI (low)** | **95% CI (high)** | ***P*** |
| **Social Class** |  |  |  |  |  |  |  |  |  |  |  |  |
| Manual | 0 | - | - | **-** | 0 | - | - | **-** | 0 | - | - | **-** |
| Non-manual | 0.595 | 0.476 | 0.743 | **<0.001** | 0.597 | 0.462 | 0.771 | **<0.001** | 0.709 | 0.549 | 0.916 | **0.008** |
| **Area deprivation*** |  |  |  |  |  |  |  |  |  |  |  |  |
| More deprived | 0 | - | - | **-** | 0 | - | - | **-** | 0 | - | - | **-** |
| Less deprived | 0.617 | 0.499 | 0.763 | **<0.001** | 0.571 | 0.447 | 0.730 | **<0.001** | 0.806 | 0.630 | 1.031 | **0.087** |
| **Home ownership** |  |  |  |  |  |  |  |  |  |  |  |  |
| Renter | 0 | - | - | **-** | 0 | - | - | **-** | 0 | - | - | **-** |
| Owner | 0.567 | 0.455 | 0.705 | **<0.001** | 0.596 | 0.467 | 0.760 | **<0.001** | 0.747 | 0.582 | 0.959 | **0.022** |
| **Employment** |  |  |  |  |  |  |  |  |  |  |  |  |
| Not employed | - | - | - | **-** | 0 | - | - | **-** | 0 | - | - | **-** |
| Employed | - | - | - | **-** | 0.631 | 0.490 | 0.831 | **<0.001** | 0.872 | 0.682 | 1.114 | **0.272** |
| **Limiting Longstanding Illness** |  |  |  |  |  |  |  |  |  |  |  |  |
| Yes | 0 | - | - | **-** | 0 | - | - | **-** | 0 | - | - | **-** |
| No | 1.101 | 0.769 | 1.577 | **0.599** | 0.857 | 0.633 | 1.161 | **0.319** | 0.999 | 0.766 | 1.304 | **0.994** |
| **Lung function†** |  |  |  |  |  |  |  |  |  |  |  |  |
| Low FEV1 | 0 | - | - | **-** | 0 | - | - | **-** | 0 | - | - | **-** |
| High FEV1 | 0.689 | 0.406 | 1.169 | **0.167** | 0.752 | 0.491 | 1.151 | **0.752** | 0.986 | 0.768 | 1.265 | **0.909** |
| **Blood pressure‡** |  |  |  |  |  |  |  |  |  |  |  |  |
| High | 0 | - | - | **-** | 0 | - | - | **-** | 0 | - | - | **-** |
| Low | 1.080 | 0.613 | 1.902 | **0.790** | 0.825 | 0.617 | 1.102 | **0.192** | 1.543 | 1.177 | 2.023 | **0.002** |
| **Self-rated health** |  |  |  |  |  |  |  |  |  |  |  |  |
| Poor/Fair | 0 | - | - | **-** | 0 | - | - | **-** | 0 | - | - | **-** |
| Good/Excellent | 0.611 | 0.368 | 1.014 | **0.056** | 0.740 | 0.558 | 0.981 | **0.036** | 0.981 | 0.753 | 1.277 | **0.885** |
| **Male** |  |  |  |  |  |  |  |  |  |  |  |  |
| Yes | 0 | - | - | **-** | 0 | - | - | **-** | 0 | - | - | **-** |
| No | 0.721 | 0.584 | 0.890 | **0.002** | 1.286 | 1.009 | 1.638 | **0.042** | 1.528 | 1.189 | 1.965 | **0.001** |
|  |  |  |  |  |  |  |  |  |  |  |  |  |

* Less deprived = Depcat ≤ 5. More deprived = Depcat >5

† FEV1 = Forced expiratory Volume in one second. FEV1 is a measure of lung function where higher values represent better lung functioning. Low FEV1 = worst quintile of FEV1 values

‡ Low blood pressure = worst quintile of diastolic blood pressure values
