# Supplementary material for: Is Telomere Length Socially Patterned? Evidence from the West of Scotland Twenty-07 Study
Source: PLoS One. 2012 Jul 23;7(7):e41805. doi: 10.1371/journal.pone.0041805 (PMC3402400; doi:10.1371/journal.pone.0041805)
Supplement: Table S4 — Significance values for the sex interaction term in the main analysis GLMs. (DOCX) [file pone.0041805.s004.docx]

**Table S4** Significance values for the sex interaction term in the main analysis GLMs

|  | **1970s** | **1950s** | **1930s** |
| --- | --- | --- | --- |
|  | ***P*** | ***P*** | ***P*** |
|  |  |  |  |
| **Social Class** | 0.100 | 0.221 | 0.102 |
| **Home Tenure** | 0.770 | 0.415 | 0.190 |
| **Income (equivalised quintiles)** | 0.530 | 0.291 | 0.273 |
| **Income (£ per week)** | 0.085 | 0.641 | 0.738 |
| **Area-deprivation** | 0.348 | 0.464 | 0.110 |
| **Area-deprivation (Carstairs)** | 0.295 | 0.791 | 0.289 |
| **Employment status** | <0.001 | 0.015 |  |
| **SES Ladder** | <0.001 | 0.889 | 0.007 |
|  |  |  |  |
| **Education (years)** | 0.611 | 0.360 | 0.229 |
| **Education (years – continuous)** | 0.033 | 0.564 | 0.177 |
| **Education (qualifications)** | 0.164 | 0.364 | 0.096 |
| **Parental class at 15 (male-dominated)** | 0.124 | 0.484 | 0.009 |
| **Household financial difficulties at 15** | 0.712 | 0.007 | 0.405 |
| **Family car ownership at 15** | 0.687 | 0.055 | 0.270 |
| **SES ladder** | 0.593 | 0.287 | 0.012 |
|  |  |  |  |
| **Social class mobility** | 0.487 | 0.716 | 0.279 |
| **Home tenure mobility** | 0.204 | 0.439 | 0.369 |
| **Number of waves in non-manual class** | 0.292 | 0.465 | 0.246 |
| **Number of waves as home owner** | 0.162 | 0.692 | 0.845 |
|  |  |  |  |
