# Supplementary material for: Is Telomere Length Socially Patterned? Evidence from the West of Scotland Twenty-07 Study
Source: PLoS One. 2012 Jul 23;7(7):e41805. doi: 10.1371/journal.pone.0041805 (PMC3402400; doi:10.1371/journal.pone.0041805)
Supplement: Table S5 — Estimated difference in telomere length associated with employment status, subjective social class/MacArthur ladder and years of education for the 1970s cohort. (DOCX) [file pone.0041805.s005.docx]

**Table S5** Estimated difference in telomere length* associated with employment status, subjective social class/ MacArthur ladder and years of education for the 1970s cohort†

|  | **Men** | | | | | **Women** | | | | |
| --- | --- | --- | --- | --- | --- | --- | --- | --- | --- | --- |
|  | **B‡** | **SE** | ***P*** | ***P_overall_*** | ***P_trend_*** | **B‡** | **SE** | ***P*** | ***P_overall_*** | ***P_trend_*** |
| **Employment status** |  |  |  |  |  |  |  |  |  |  |
| Employed | **0 (ref)** | - | - |  |  | **0 (ref)** | - | - |  |  |
| Caring for the home | **1.905** | 0.460 | <0.001 |  |  | **-0.630** | 0.272 | 0.021 |  |  |
| Retired | **-** | - | - |  |  | **-** | - | - |  |  |
| Unemployed | **-0.955** | 0.513 | 0.064 |  |  | **0.424** | 0.375 | 0.259 |  |  |
| Unable to work through ill health | **-0.073** | 0.424 | 0.864 |  |  | **-1.151** | 0.379 | 0.003 |  |  |
| Other | **-0.224** | 0.306 | 0.464 | <0.001 |  | **-0.553** | 0.340 | 0.105 | 0.004 |  |
|  |  |  |  |  |  |  |  |  |  |  |
| **SES Ladder** |  |  |  |  |  |  |  |  |  |  |
| 10 (highest) | **0 (ref)** | - | - |  |  | **0 (ref)** | - | - |  |  |
| 9 | **-2.279** | 0.765 | 0.003 |  |  | **-2.123** | 0.951 | 0.026 |  |  |
| 8 | **-2.252** | 0.481 | <0.001 |  |  | **-1.920** | 0.733 | 0.009 |  |  |
| 7 | **-1.948** | 0.436 | <0.001 |  |  | **-1.723** | 0.689 | 0.013 |  |  |
| 6 | **-1.483** | 0.466 | 0.002 |  |  | **-2.216** | 0.680 | 0.001 |  |  |
| 5 | **-2.388** | 0.506 | <0.001 |  |  | **-1.884** | 0.714 | 0.009 |  |  |
| 4 | **-1.710** | 0.568 | 0.003 |  |  | **-2.581** | 0.686 | <0.001 |  |  |
| 3 | **-2.152** | 0.548 | <0.001 |  |  | **-2.464** | 0.726 | 0.001 |  |  |
| 2 | **-1.933** | 0.607 | 0.002 |  |  | **-1.551** | 0.926 | 0.095 |  |  |
| 1 (lowest) | **-0.158** | 1.034 | 0.879 | <0.001 | 0.504 | **-2.573** | 0.909 | 0.005 | 0.006 | 0.040 |
|  |  |  |  |  |  |  |  |  |  |  |
| **Education (years – continuous)** | **0.011** | 0.029 | 0.698 |  |  | **0.095** | 0.029 | 0.001 |  |  |
|  |  |  |  |  |  |  |  |  |  |  |

* Telomere length measured as relative T/S ratio multiplied by 10

† Analysis samples are weighted to members of the baseline sample who were still alive at wave 5 and all analyses adjusted for plate

‡ Unstandardized regression coefficient
