# Supplementary material for: Is Telomere Length Socially Patterned? Evidence from the West of Scotland Twenty-07 Study
Source: PLoS One. 2012 Jul 23;7(7):e41805. doi: 10.1371/journal.pone.0041805 (PMC3402400; doi:10.1371/journal.pone.0041805)
Supplement: Table S6 — Estimated difference in telomere length associated with employment status and household financial difficulties at age 15 for the 1950s cohort. (DOCX) [file pone.0041805.s006.docx]

**Table S6** Estimated difference in telomere length* associated with employment status and household financial difficulties at age 15 for the 1950s cohort†

|  | **Men** | | | | | | | | **Women** | | | | | | |
| --- | --- | --- | --- | --- | --- | --- | --- | --- | --- | --- | --- | --- | --- | --- | --- |
|  | **B‡** | **SE** | ***P*** | ***P_overall_*** | ***P_trend_*** | **B‡** | | **SE** | | ***P*** | ***P_overall_*** | ***P_trend_*** |  |  |  |
| **Employment status** |  |  |  |  |  |  | |  | |  |  |  |  |  |  |
| Employed | **0 (ref)** | - | - | | |  |  | | **0 (ref)** | | | - | - |  |  |
| Caring for the home | **-0.478** | 0.643 | 0.458 |  |  | **0.234** | | 0.296 | | 0.430 |  |  |  |  |  |
| Retired | **-0.390** | 0.258 | 0.132 |  |  | **-0.642** | | 0.287 | | 0.026 |  |  |  |  |  |
| Unemployed | **-0.064** | 0.360 | 0.859 | | |  |  | | **0.323** | | | 0.643 | 0.616 |  |  |
| Unable to work through ill health | **0.742** | 0.263 | 0.005 | | |  |  | | **-0.422** | | | 0.230 | 0.068 |  |  |
| Other | **-0.814** | 0.262 | 0.002 | | | 0.001 |  | | **-0.393** | | | 0.409 | 0.337 | 0.041 |  |
|  |  |  |  | | |  |  | |  | | |  |  |  |  |
| **Household financial difficulties at 15** |  |  |  | | |  |  | |  | | |  |  |  |  |
| Very well off | **0 (ref)** | - | - | | |  |  | | **0 (ref)** | | | - | - |  |  |
| Quite well off | **4.204** | 0.001 | <0.001 | | |  |  | | **-0.122** | | | 0.804 | 0.879 |  |  |
| Usually had just enough money | **4.155** | 0.226 | <0.001 | | |  |  | | **0.129** | | | 0.787 | 0.870 |  |  |
| Sometimes short of money | **4.343** | 0.254 | <0.001 | | |  |  | | **0.105** | | | 0.788 | 0.894 |  |  |
| Often short of money | **4.127** | 0.350 | <0.001 | | | <0.001 | 0.739 | | **-0.452** | | | 0.820 | 0.582 | 0.231 | 0.742 |
|  |  |  |  | | |  |  | |  | | |  |  |  |  |

* Telomere length measured as relative T/S ratio multiplied by 10.

† Analysis samples are weighted to members of the baseline sample who were still alive at wave 5 and all analyses adjusted for plate

‡ Unstandardized regression coefficient
