# Supplementary material for: Is Telomere Length Socially Patterned? Evidence from the West of Scotland Twenty-07 Study
Source: PLoS One. 2012 Jul 23;7(7):e41805. doi: 10.1371/journal.pone.0041805 (PMC3402400; doi:10.1371/journal.pone.0041805)
Supplement: Table S7 — Estimated difference in telomere length associated with subjective social class/MacArthur ladder at ages 15 and 75, and parental social class at age 15 for the 1930s cohort. (DOCX) [file pone.0041805.s007.docx]

**Table S7** Estimated difference in telomere length* associated with subjective social class/ MacArthur ladder at ages 15 and 75, and parental social class at age 15 for the 1930s cohort†

|  | **Men** | | | | | **Women** | | | | |
| --- | --- | --- | --- | --- | --- | --- | --- | --- | --- | --- |
|  | **B‡** | **SE** | ***P*** | ***P_overall_*** | ***P_trend_*** | **B‡** | **SE** | ***P*** | ***P_overall_*** | ***P_trend_*** |
| **SES Ladder** |  |  |  |  |  |  |  |  |  |  |
| 10 (highest) | **0 (ref)** | - | - |  |  | **0 (ref)** | - | - |  |  |
| 9 | **0.668** | 1.439 | 0.643 |  |  | **1.064** | 0.677 | 0.117 |  |  |
| 8 | **0.690** | 1.183 | 0.560 |  |  | **0.175** | 0.756 | 0.817 |  |  |
| 7 | **0.721** | 1.137 | 0.526 |  |  | **0.441** | 0.735 | 0.549 |  |  |
| 6 | **0.554** | 1.176 | 0.638 |  |  | **0.498** | 0.724 | 0.492 |  |  |
| 5 | **0.721** | 1.169 | 0.538 |  |  | **0.727** | 0.732 | 0.321 |  |  |
| 4 | **1.040** | 1.198 | 0.386 |  |  | **1.099** | 0.735 | 0.136 |  |  |
| 3 | **0.589** | 1.196 | 0.623 |  |  | **0.074** | 0.787 | 0.925 |  |  |
| 2 | **0.618** | 1.196 | 0.606 |  |  | **0.051** | 0.979 | 0.958 |  |  |
| 1 (lowest) | **0.052** | 1.224 | 0.966 | 0.828 | 0.655 | **2.926** | 0.845 | 0.001 | <0.001 | 0.065 |
|  |  |  |  |  |  |  |  |  |  |  |
| **Parental social class at 15** |  |  |  |  |  |  |  |  |  |  |
| I (Professional etc occupations) | **0 (ref)** | - | - |  |  | **0 (ref)** | - | - |  |  |
| II (Managerial and technical occupations) | **-0.962** | 0.528 | 0.070 |  |  | **-0.516** | 0.347 | 0.139 |  |  |
| III – NM (Non-manual skilled occupations) | **-0.947** | 0.520 | 0.070 |  |  | **0.588** | 0.380 | 0.123 |  |  |
| III – M (Manual skilled occupations) | **-1.174** | 0.500 | 0.020 |  |  | **0.041** | 0.319 | 0.898 |  |  |
| IV (Partly-skilled occupations) | **-1.943** | 0.703 | 0.006 |  |  | **0.034** | 0.455 | 0.941 |  |  |
| V (Unskilled occupations) | **-1.687** | 0.670 | 0.012 | 0.063 | 0.004 | **0.841** | 0.493 | 0.089 | 0.002 | 0.028 |
|  |  |  |  |  |  |  |  |  |  |  |
| **SES Ladder** |  |  |  |  |  |  |  |  |  |  |
| 10 (highest) | **0 (ref)** | - | - |  |  | **0 (ref)** | - | - |  |  |
| 9 | **0.432** | 0.551 | 0.434 |  |  | **-0.756** | 0.834 | 0.365 |  |  |
| 8 | **-0.797** | 0.563 | 0.158 |  |  | **0.280** | 0.847 | 0.741 |  |  |
| 7 | **-1.148** | 0.635 | 0.072 |  |  | **-0.597** | 0.831 | 0.473 |  |  |
| 6 | **-1.347** | 0.514 | 0.009 |  |  | **-0.386** | 0.823 | 0.639 |  |  |
| 5 | **-0.254** | 0.546 | 0.643 |  |  | **-0.510** | 0.818 | 0.534 |  |  |
| 4 | **-0.700** | 0.529 | 0.187 |  |  | **0.302** | 0.839 | 0.719 |  |  |
| 3 | **-1.041** | 0.528 | 0.050 |  |  | **-0.405** | 0.853 | 0.635 |  |  |
| 2 | **-0.589** | 0.557 | 0.291 |  |  | **-0.080** | 0.815 | 0.922 |  |  |
| 1 (lowest) | **-1.264** | 0.824 | 0.126 | <0.001 | 0.497 | **-1.902** | 0.816 | 0.020 | <0.001 | 0.843 |

* Telomere length measured as relative T/S ratio multiplied by 10.

† Analysis samples are weighted to members of the baseline sample who were still alive at wave 5 and all analyses adjusted for plate

‡ Unstandardized regression coefficient
