# Supplementary material for: Is Telomere Length Socially Patterned? Evidence from the West of Scotland Twenty-07 Study
Source: PLoS One. 2012 Jul 23;7(7):e41805. doi: 10.1371/journal.pone.0041805 (PMC3402400; doi:10.1371/journal.pone.0041805)
Supplement: Table S8 — Bonferroni adjusted P-values according to the three key life stages where SES was measured. (DOCX) [file pone.0041805.s008.docx]

**Table S8** Bonferroni adjusted P-values according to the three key life stages where SES was measured

| **Basic formula** |  |  |
| --- | --- | --- |
| Adjusted *P* value | **=** | ***P* / x** |
|  | **=** | **0.05 / x** |
| **Contemporaneous SES (x = 8)** |  |  |
| Adjusted *P* value | **=** | **0.05 / 8** |
|  | **=** | **0.006** |
|  |  |  |
| **Education and childhood SES (x = 7)** |  |  |
| Adjusted *P* value | **=** | **0.05 / 7** |
|  | **=** | **0.007** |
|  |  |  |
| **SES over time (x = 4)** |  |  |
| Adjusted *P* value | **=** | **0.05 / 4** |
|  | **=** | **0.013** |

x = number of comparisons / SES variables
